# *CACNA1A* Genetic Variants and Their Potential Involvement in Migraine Pathogenesis

**DOI:** 10.3390/ijms26168083

**Published:** 2025-08-21

**Authors:** Oliwia Szymanowicz, Bartosz Słowikowski, Joanna Poszwa, Ulyana Goutor, Małgorzata Wiszniewska, Paweł P. Jagodziński, Wojciech Kozubski, Jolanta Dorszewska

**Affiliations:** 1 Laboratory of Neurobiology, Department of Neurology, Poznan University of Medical Sciences, 60-355 Poznan, Poland; 2 Doctoral School, Poznan University of Medical Sciences, 60-812 Poznan, Poland; 3 Department of Biochemistry and Molecular Biology, Poznan University of Medical Sciences, 60-781 Poznan, Poland; 4 Faculty of Health, Care Stanislaw Staszic University of Applied Sciences in Pila, 64-920 Pila, Poland; mpwisz@gmail.com; 5 Department of Neurology, Specialistic Hospital in Pila, 64-920 Pila, Poland; 6 Chair and Department of Neurology, Poznan University of Medical Sciences, 60-355 Poznan, Poland

**Keywords:** genetic variants, *CACNA1A* gene, pathogenesis, migraine

## Abstract

Migraine is a prevalent neurological disorder that affects over 1 billion individuals worldwide. The pathogenesis of migraine remains incompletely understood, though evidence suggests a multifactorial etiology involving genetic factors. The *CACNA1A* gene has been implicated in rare forms of Familial Hemiplegic Migraine (FHM). This study aimed to investigate the role of *CACNA1A* variants in individuals with and without a family history of migraine. We genotyped 150 subjects (100 migraine patients: 50 with migraine without aura (MO), 50 with migraine with aura (MA) and 50 controls) for six *CACNA1A* variants using Sanger sequencing. Statistical analyses were performed in Statistica (*p* < 0.05). The CADD v1.7 model was used to assess the potential pathogenicity of novel variants. Three variants described in databases (*rs10405121*, *rs894252513*, and *rs1012663275*) and three novel variants (*ch19:13228374 G* > *C*, *ch19:13228428 G* > *C*, and *ch19:13228348 A* > *T*) were identified. The *rs10405121* variant was associated with both migraine types, with the homozygous AA genotype exclusively found in familial cases. Abnormal genotype of *rs894252513* and *rs1012663275* were detected only in familial cases with MO. The novel variants were observed exclusively in patients with a family history of migraine, suggesting their potential relevance to inherited migraine pathogenesis. Novel variants may contribute to migraine pathogenesis by altering calcium channel function and lowering the threshold for cortical spreading depression (CSD).

## 1. Introduction

Migraine is one of the most prevalent and disabling neurological disorders worldwide, affecting approximately 40% of the global population [1]. According to the Global Burden of Disease Study, migraine is the second leading cause of years lived with disability (YLDs) globally, highlighting its significant impact on public health. It is estimated that over 1 billion individuals suffer from migraine, with women being three times more likely to experience the condition than men [2,3]. The prevalence peaks during the most productive years of life, between the ages of 25 and 55, resulting in substantial personal, societal, and economic consequences [4].

The burden of migraine extends beyond episodic headaches, severely impairing quality of life [5]. Migraines are clinically classified into two primary subtypes based on the presence or absence of aura: migraine with aura (MA) and migraine without aura (MO). MO attacks are characterized by intense pain and accompanying symptoms, such as nausea, vomiting, and sensory sensitivities, which often leave patients unable to function during episodes. MA, on the other hand, is characterized by transient neurological disturbances that precede or accompany the headache phase. Aura symptoms typically develop gradually over 5–60 min and can include visual phenomena, such as flashing lights, zigzag patterns, or temporary vision loss. Other aura symptoms may involve sensory changes, such as numbness or tingling, and, less commonly, language disturbances or motor weakness [6,7]. This wide range of symptoms is associated with increased rates of anxiety, depression, and reduced overall quality of life.

Diagnosing migraine remains a significant challenge due to its complex and varied presentation [8]. Many patients experience delays in receiving an accurate diagnosis because migraine is a multifactorial disease with environmental, biochemical, hormonal, immunological, and genetic components [9]. The pathogenesis of migraine involves impaired neuronal transmission, increased neuropeptide activity (e.g., calcitonin gene-related peptide (CGRP) and pituitary adenylate cyclase-activating polypeptide (PACAP)), ion channel dysfunction, neuroinflammatory processes, and disturbances in serotonergic systems [10]. Additionally, the most reported risk factors for migraines include genetic predisposition, female gender, young age, a positive family history, hormonal imbalances (e.g., estrogen fluctuations), stress, vitamin deficiencies (including vitamin D), and environmental factors such as weather changes, noise, or strong sensory stimuli. These factors not only increase the risk of migraines but also influence the frequency and severity of attacks. For example, vitamin D plays a role in immune regulation, neuroprotection, and calcium homeostasis, and its deficiency may contribute to symptom exacerbation [11,12]. Understanding the complex mechanisms underlying migraine is essential for improving diagnostic accuracy and therapeutic strategies.

Despite its high prevalence and significant impact, the pathogenesis of migraine remains incompletely understood and is largely based on theoretical models. Among these, cortical spreading depression (CSD) has been extensively studied and is considered central to migraine development [13].

CSD is a key pathophysiological mechanism implicated in migraine, particularly in cases involving aura. It is defined as a slow, self-propagating wave of neuronal and glial depolarization followed by a period of suppressed cortical activity [14].

The connection between CSD and the *CACNA1A* gene is rooted in the gene’s critical role in regulating neuronal excitability and synaptic transmission. The *CACNA1A* gene encodes the α1A subunit of the P/Q-type voltage-gated calcium channels, which are highly expressed in cortical neurons [15]. P/Q-type channels are high-threshold channels with rapid activation and inactivation, allowing precise temporal control of synaptic transmission. These channels activate in response to membrane depolarization, causing a conformational change in the channel protein, opening its pore, and enabling a rapid influx of Ca2+ ions from the extracellular space into the cytoplasm [16]. This calcium influx is rapid and transient, but it plays a key role as a secondary signaling molecule—Ca^2+^ ions bind to synaptic vesicle proteins, such as synaptotagmin, initiating vesicle fusion with the presynaptic membrane and neurotransmitter release into the synaptic cleft [16,17].

While the exact triggers of CSD remain under investigation, factors such as cortical excitability, genetic predisposition, and external stimuli (e.g., stress, sleep disturbances, or dietary factors) are thought to influence its initiation [18]. The genetic basis for CSD is supported by its strong association with familial hemiplegic migraine (FHM), where mutations in genes, such as *CACNA1A*, *ATP1A2*, and *SCN1A*, alter ion channel function, predisposing the brain to hyperexcitability [19,20]. In familial hemiplegic migraine type 1 (FHM1), mutations in the *CACNA1A* gene lead to an increased influx of calcium ions (Ca^2+^) through P/Q-type voltage-gated calcium channels, resulting in excessive neuronal excitability and a lowered threshold for CSD. This mechanism is characteristic of migraine with hemiplegic aura [20,21].

Evidence suggests that CSD may also play a role in common forms of migraine, such as MA [22]. Animal models of FHM1 further demonstrate that *CACNA1A* mutations enhance cortical excitability and prolong the duration of CSD, contributing to both the aura and the subsequent activation of the trigeminovascular system, which leads to headache [23]. This relationship between *CACNA1A* and CSD highlights the broader role of ion channel dysfunction in migraine [24].

Mutations in *CACNA1A* are known to alter the functional properties of calcium channels, leading to either gain-of-function or loss-of-function effects [25]. In the context of migraine, gain-of-function mutations typically increase calcium channel activity, resulting in excessive neuronal excitability and a lowered threshold for initiating CSD [19]. This heightened neuronal excitability predisposes cortical neurons to the ionic and metabolic imbalances necessary to trigger the self-propagating wave of depolarization that is characteristic of CSD [26].

However, it is worth noting that different mutations in *CACNA1A* gene may lead to different functional effects (Table 1). For example, in spinocerebellar ataxia type 6 (SCA6), mutations in the *CACNA1A* gene cause the expansion of CAG repeats, which results in the formation of abnormal proteins with long glutamine sequences [27]. This leads to the progressive degeneration of Purkinje cells in the cerebellum, resulting in symptoms such as ataxia and dysarthria [28].

Along with other factors involved in migraine pathogenesis, ion channel dysfunction can be considered a key contributor [29,30]. This perspective highlights the genetic basis of migraine as a central factor in its development. Our study aims to explore this connection by investigating genetic variants of the *CACNA1A* gene, with the goal of identifying specific alterations that may contribute to migraine susceptibility and enhancing our understanding of its molecular mechanisms.

## 2. Results

We identified three previously described variants of the *CACNA1A* gene, namely *rs10405121*, *rs894252513*, *rs1012663275*, and three novel variants not found in genetic databases: *ch19:13228374 G* > *C*, *ch19:13228428 G* > *C*, *ch19:13228348 A* > *T*. It was observed that individual genotypes varied depending on the clinical features of the patients (Table 2). All the variants identified in this study, including both known and novel ones, are located within the intronic regions of the *CACNA1A* gene.

The *rs10405121* variant (*ch19:13228314 G* > *A*, GRCh38.p14) involves the substitution of the normal G allele with the abnormal A allele [31]. This genetic alteration may affect the functional properties of the *CACNA1A* gene. The abnormal homozygous AA genotype was detected in 14 migraine patients (17%) (10 with MA and 4 with MO) with a family history, and in 6 migraine patients (33%) (5 with MA and 1 with MO) without a family history, as well as in 9 controls (18%). The heterozygous GA genotype was found in 35 migraine patients (43%) (10 with MA and 25 with MO) with a family history, and in 5 migraine patients (28%) (2 with MA and 3 with MO) without a family history, as well as in 25 controls (50%). The normal homozygous GG genotype was observed in 40 migraine patients (40%) and 16 controls (32%).

**Figure 1:**
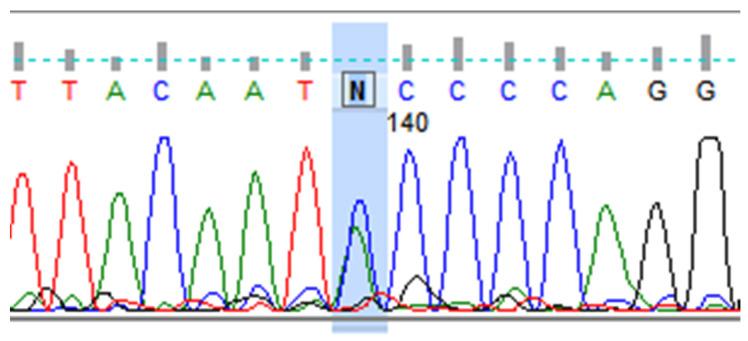
Heterozygous variant in the *CACNA1A* gene, *rs894252513*. DNA sequence analysis using the Sanger method, showing the letter “N”, indicates an unidentified nitrogenous base at position 140 from the start of the sequence. “N” is the heterozygous C > A variant (*rs894252513*) in the *CACNA1A* gene. A double peak in the chromatogram is visible (marked in blue), indicating the presence of two alleles (C and A) at the nucleotide position *ch:19:13228392*, (GRCh38.p14).

For the *rs894252513* genetic variant (*ch:19:13228392 C* > *A*, GRCh38.p14), the normal C allele is replaced by the abnormal A allele [32] (Figure 1.). The abnormal heterozygous CA genotype was exclusively detected in one migraine patient (1%) with a family history and MO. This variant was absent in patients without a family history and in the control group. The normal homozygous CC genotype was present in both migraine patients with (99%) and without (100%) a family history, as well as in controls (100%). Although the frequency of the abnormal heterozygous CA genotype was low, its exclusive presence in familial cases suggests its potential relevance to migraine.

The *rs1012663275* genetic variant (*ch19:13228397 A* > *C*, GRCh38.p14) involves the substitution of the normal A allele with the abnormal C allele [33]. The abnormal homozygous CC genotype was identified in one migraine patient (1%) with a family history and MO. Like *rs894252513*, this variant was absent in non-familial migraine cases (0%), further highlighting its potential link to genetic predisposition in MO. The heterozygous AC genotype was observed only in 5 migraine patients (6%) (2 with MA and 3 with MO) with a family history, and in 14 controls (28%). The normal homozygous AA genotype was found in 76 migraine patients (93%) (34 with MA and 42 with MO) with a family history, in 18 migraine patients (100%) (14 with MA and 4 with MO) without a family history, and in 34 controls (68%).

In contrast to the variants of the *CACNA1A* gene reported in genetic databases, three novel variants of the *CACNA1A* gene were identified: *ch19:13228374 G* > *C*, *ch19:13228428 G* > *C*, and *ch19:13228348 A* > *T*. The first, *ch19:13228374 G* > *C*, was found in 6 patients (7%) with a family history of migraine and a young age of onset (6–20 years), presenting two different types of migraine: MA (*n* = 1) and MO (*n* = 5). The ch19:13228428 G > C variant was detected in 12 patients (15%) (4 with MA and 8 with MO) with a family history. These patients also experienced early onset of migraine (average age 16 ± 7), but MA patients had an earlier onset (average age 15 ± 7), with the youngest patient experiencing migraine since the age of 5. The final variant, *ch19:13228348 A* > *T*, was identified exclusively in two patients with a family history and MO. This novel *CACNA1A* variant was absent in the control group, suggesting a potential role in migraine pathogenesis; however, further research on larger cohorts is needed to confirm this association.

A comparative analysis of genotype frequencies between migraine patients (both with aura and without aura) and controls revealed statistically significant associations (Table 3). The *rs1012663275* variant showed the strongest association, with two genotypes (AC, AA) differing significantly between migraine patients and controls (*p* < 0.01).

In contrast, rare or novel variants such as *rs1012663275*, *ch19:13228374 G* > *C*, *ch19:13228428 G* > *C*, and *ch19:13228348 A* > *T* were more variably distributed in our study group. For example, the AT genotype of the novel A > T substitution (*ch19:13228348*) was observed exclusively in patients with familial MO and was absent in the control group. Although statistical testing was not possible due to the small sample size, this variant may represent a rare migraine-associated mutation.

To further investigate their potential biological impact, in silico analysis using the CADD v1.7 model (GRCh38) was performed (Table 4). The *ch19:13228428 G* > *C* variant showed a high PHRED score of 17.06, suggesting a potentially deleterious effect. In contrast, *ch19:13228348 A* > *T* and *ch19:13228374 G* > *C* had lower scores (6.464 and 0.921, respectively), indicating that they may have a benign or only mildly regulatory role. These findings suggest that some of the newly identified intronic variants could exert functional effects relevant to migraine pathophysiology. However, due to limited sample sizes, statistical significance could not be formally established for these observations, and further research with larger cohorts is needed to validate these results.

## 3. Discussion

Migraine is a highly polygenic and multifactorial disorder [34,35]. In addition to the *ATP1A2* and *SCN1A* genes, the *CACNA1A* gene has also been implicated in its pathogenesis [36]. All of these genes encode ion channels which are critical for maintaining neuronal homeostasis. Variants in these genes, including *CACNA1A*, have been associated with rare subtypes of migraine, such as familial hemiplegic migraine, where mutations alter cortical excitability and predispose individuals to CSD [37]. In a knock-in mouse model with the human mutation associated with FHM-1 (*CACNA1A* R192Q), increased Ca(v)2.1 current density in cerebellar neurons and an enhanced velocity of CSD responsible for the aura symptom in migraine was demonstrated [23]. The association of the *CACNA1A* gene with migraine is the subject of ongoing research, with new genetic variants in this gene regularly reported in the literature [38].

In our study, we analyzed the *CACNA1* gene and identified three previously reported variants (*rs10405121*, *rs894252513*, and *rs1012663275*) and three novel variants (*ch19:13228374 G* > *C*, *ch19:13228428 G* > *C*, and *ch19:13228348 A* > *T*) in migraine patients. Notably, individual genotypes varied depending on the type of migraine and family history, underscoring the genetic complexity of this disorder.

The rs10405121 variant of the *CACNA1A* gene showed distinct genotype distributions between migraine patients and controls. The abnormal homozygous AA genotype was observed in migraine patients (*n* = 20) twice as often as in controls (*n* = 9). Interestingly, the AA genotype was more frequently found in patients with migraine with aura (MA). For each genotype (AA, GA and GG) of the *rs10405121* variant, it was observed that it appeared more often in patients with MA. This supports the hypothesis that this variant may contribute to the fundamental mechanisms of migraine pathogenesis, such as neuronal excitability and susceptibility to CSD. Additionally, a 2022 study suggests that this variant is specific to MA [39,40].

In contrast, the abnormal CA homozygous genotype of the *rs894252513* variant and the abnormal CC homozygous genotype of the *rs1012663275* variant were exclusively detected in patients with familial migraine and absent in non-familial cases. The absence of these variants in non-familial cases suggests their potential role in genetic predisposition to familial migraine. This aligns with previous studies linking rare genetic variants in ion channel-related genes, such as *ATP1A2* and *SCN1A* [35], to familial forms of migraine, further supporting the hypothesis of migraine as a channelopathy [38,41,42].

The identification of three novel *CACNA1A* variants (*ch19:13228374 G* > *C*, *ch19:13228428 G* > *C*, and *ch19:13228348 A* > *T*) may enhance our understanding of the gene’s role in migraine pathophysiology. In our study, this is the first time these variants have been linked to migraine. Notably, two of these novel variants (*ch19:13228374 G* > *C* and *ch19:13228348 A* > *T*) were identified exclusively in patients with a family history of migraine, and were more commonly associated with MO, suggesting a potential genetic component linked specifically to this type of migraine. The absence of these variants in the control group strongly supports their potential role as risk factors for familial migraine susceptibility. Additionally, in silico analysis using the CADD v1.7 model revealed divergent predicted impacts among the novel *CACNA1A* variants. Specifically, the *ch19:13228428 G* > *C* variant showed a high PHRED score (17.06), indicating a potential deleterious effect and supporting its involvement in migraine susceptibility. In contrast, the *ch19:13228348 A* > *T* and *ch19:13228374 G* > *C* variants had lower scores (6.464 and 0.921, respectively), suggesting limited or no functional impact. These findings underscore the need for further studies to explore the functional significance of these variants, particularly in relation to neuronal excitability and CSD, both of which are strongly influenced by *CACNA1A* dysfunction.

The *ch19:13228374 G* > *C* variant was found in six patients with a family history of migraine and a young age of onset (6–20 years), with two different migraine types: MA (*n* = 1) and MO (*n* = 5). The identification of this variant in patients with different migraine phenotypes highlights the complexity of migraine pathogenesis and suggests that individual genetic alterations might predispose to migraine in general, rather than exclusively to one subtype.

The *ch19:13228428 G* > *C* variant, detected in twelve familial migraine patients, exhibited intriguing variability in clinical presentation, including both MA and MO subtypes. Notably, migraine onset occurred relatively early, with an average age of 16 ± 7 years. Interestingly, patients with MA carrying this variant had a significantly earlier onset (ages 5 and 15) compared to those with MO, indicating a possible genotype-phenotype correlation. This variant was also present in two patients with no family history, both of whom had only the MA subtype.

The *ch19:13228348 A* > *T* variant was identified exclusively in two familial MO cases, suggesting a potential subtype-specific effect. The consistent MO phenotype among patients with this variant may indicate a more targeted pathogenic mechanism.

The identification of *CACNA1A* variants strengthens the hypothesis that migraine may be a channelopathy. Mutations in *CACNA1A* are known to alter calcium channel function, leading to neuronal hyperexcitability and a lowered threshold for initiating CSD, a key mechanism in migraine, particularly in cases with aura. A 2016 study by Hu et al. [43] found that *CACNA1A* variants, such as *rs8182538*, were associated with increased diastolic blood pressure and elevated hypertension risk, suggesting a role in vascular regulation. This is relevant given the neurovascular nature of migraine, particularly in subtypes like migraine with aura. Although the variants identified in our study differ from those in Hu’s research, their potential to influence both neuronal and vascular pathways should not be excluded. Therefore, our findings should be considered within the context of broader physiological mechanisms, and future studies should investigate the links between *CACNA1A* variants, migraine, and vascular traits.

## 4. Materials and Methods

This study was designed to investigate the role of *CACNA1A* gene variants in migraine pathogenesis. Patients were recruited based on criteria from the International Classification of Headache Disorders, 3rd edition (ICHD-3). Migraine patients were diagnosed by experienced neurologists in the Clinical Hospital of Heliodor Święcicki in Poznan. Inclusion criteria consisted of a confirmed diagnosis of either MA or MO, according to ICHD-3 guidelines, along with signed informed consent for participation and genetic testing. Patients were excluded if they had other primary or secondary headache disorders or comorbid neurological diseases (such as epilepsy or a history of traumatic brain injury). Participants volunteered for genetic testing at the Neurobiology Laboratory, Department of Neurology, Poznan University of Medical Sciences (PUMS) to detect genetic variants in the *CACNA1A* gene. Six genetic variants were examined: three previously described in the literature (*rs10405121*, *rs894252513*, *rs1012663275*) and three novel variants (*ch19:13228374 G* > *C*, *ch19:13228428 G* > *C*, and *ch19:13228348 A* > *T*). Genetic variations in the *CACNA1A* gene were analyzed in 150 subjects (47 females with MO, 45 females with MA, 44 females in the control group; 3 males with MO, 5 males with MA and 6 males as controls). The final study group included 150 individuals: 100 migraine patients (82 with a family history and 18 without) and 50 controls (Table 5).

This study was approved by the PUMS Local Bioethics Committee (No. 931/17 of 4 December 2017, with extension No. 971/22 of 8 December 2022, valid until 2025 and No. 16/25 of 9 January 2025, valid until 2028).

Venous blood (EDTA) was collected and stored at −80 °C. Genomic DNA was isolated using the Blood Mini Plus kit (A&A Biotechnology, Gdańsk, Poland). The genetic variants of the *CACNA1A* gene were analyzed via high-resolution melt analysis (HRMA) using the CFX Connect™ Real-Time system BR002503. Genomic DNA was amplified using HRMA with EvaGreen, an intercalating dye, and the SsoFast™ EvaGreen^®^ Supermix (Bio-Rad, Hercules, CA, USA). Primers for HRMA were designed using publicly available databases, based on the published genome sequence of the *CACNA1A* gene *rs10405121* (F-5’GTTGGTCACGTTCTCTGGT3’; R-5’CTTAGCTGAAGCTGCCCATC3’). Temperature gradient PCR (MJ Mini™ Gradient Thermal Cycler, Bio-Rad, Hercules, CA, USA) was initially performed for selected primer pairs to optimize annealing to the DNA template.

HRMA results were validated at an independent facility by Sanger sequencing (Applied Biosystems HITACHI, Santa Clara, CA, USA). Data were analyzed using FinchTV 1.4.0, and confirmed with databases from the National Library of Medicine (NIH, Bethesda, MD, USA) (https://www.nlm.nih.gov/, accessed on 15 July 2025), Ensembl.org (The Ensembl Genomes Project, Cambridge, UK) (https://www.ensembl.org/index.html, accessed on 15 July 2025), and Varsome (Saphetor SA, Lausanne, Switzerland) (https://varsome.com/, accessed on 15 July 2025) databases. Statistical analyses were conducted in Statistica 13.3 (*p* < 0.05). Comparisons of genotype frequencies were conducted using Fisher’s exact test. Novel intronic variants were evaluated in silico using CADD v1.7 (PHRED > 10 considered potentially functional).

## 5. Conclusions

To date, genetic variants of *CACNA1A* have been associated with several neurological disorders, including FHM1, epilepsy, cerebellar ataxia, dystonia, and cerebellar atrophy. Our studies suggest that these variants are likely also linked to migraine, both with and without a family history, as well as to early-onset migraine and migraine with or without aura. However, confirmation of these observations requires further research.

In summary, this study enhances our understanding of the clinical course and genetic basis of migraine and underscores the potential of the *CACNA1A* gene as a target for future research and therapeutic development.

## Figures and Tables

**Table 1:** *CACNA1A* gene mutation and different clinical effects.

Mutation	Disease	Mechanism	Clinical Symptoms
p.Thr501Met	Familial Hemiplegic Migraine Type 1 (FHM1)	Increased calcium ion influx, lowered CSD ^1^ threshold	Migraine with aura, hemiplegia, neuronal hyperexcitability
p.Arg192Gln	FHM1	Increased activity of calcium channels	Severe migraine attacks with aura, occasionally coma
p.Ser218Leu	FHM1	Significant increase in calcium channel permeability	Severe migraine attacks, aura, ataxia
p.Gly293Arg	FHM1	Increased calcium current, lowered neuronal excitability threshold	Severe migraines, sometimes with neurological disturbances
p.Ala454Thr	FHM1	Increased calcium channel activity	Hemiplegic migraine attacks, aura
c.2494C > T (p.Arg831Cys)	Episodic Ataxia Type 2 (EA2)	Decreased calcium ion influx through P/Q channels	Ataxia episodes, vertigo, nystagmus
c.6793C > T (p.Arg2265Trp)	EA2	Reduced calcium channel activity	Episodic ataxia triggered by stress or exertion
CAG repeat expansion	Spinocerebellar Ataxia Type 6 (SCA6)	Formation of abnormal proteins with long glutamine sequences, degeneration of Purkinje cells	Progressive ataxia, dysarthria, nystagmus

^1^ Cortical spreading depression.

**Table 2:** Distribution of *CACNA1A* gene variants among migraine patients and controls.

Variant	Genotype	Patients with Family History	Patients Without Family History	Controls	Clinical Features
*rs10405121*	AA (abnormal)	14 (17%) 10 MA ^1^, 4 MO ^2^	6 (33%) 5 MA, 1 MO	9 (18%)	Found more often in MA.
	GA (heterozygous)	35 (43%) 10 MA, 25 MO	5 (28%) 2 MA, 3 MO	25 (50%)	Present in both migraine patients and controls.
	GG (normal)	33 (40%) 16 MA, 17 MO	7 (39%) 7 MA, 0 MO	16 (32%)	Present in both migraine patients and controls.
*rs894252513*	CA (abnormal)	1 (1%) MO	0 (0%)	0 (0%)	Found exclusively in familial MO cases.
	CC (normal)	81 (99%) 36 MA, 45 MO	18 (100%) 14 MA, 4 MO	50 (100%)	Present in both migraine patients and controls.
*rs1012663275*	CC (abnormal)	1 (1%) MO	0 (0%)	2 (4%)	Found only in a familial MO case.
	AC (heterozygous)	5 (6%) 2 MA, 3 MO	0 (0%)	14 (28%)	Present more often in controls.
	AA (normal)	76 (93%) 34 MA, 42 MO	18 (100%) 14 MA, 4 MO	34 (68%)	Present more often in familial MA and controls.
*ch19:13228374 G* > *C*	-	6 (7%) 1 MA, 5 MO	0 (0%)	14 (28%)	Found in migraine patients with a young age of onset (6–20 years).
*ch19:13228428 G* > *C*	-	12 (15%) 4 MA, 8 MO	2 (11%) 2 MA, 0 MO	16 (32%)	Novel variant observed in a familial case with differences in migraine type.
*ch19:13228348 A* > *T*	-	2 (2%) MO	0 (0%)	0 (0%)	Novel variant exclusively associated with familial MO cases.

^1^ Migraine with aura. ^2^ Migraine without aura.

**Table 3:** Statistical comparison of *CACNA1A rs10405121*, *rs1012663275* and *rs894252513* variants between migraine patients and controls using Fisher’s test.

Genetic Variant	Genotype	Migraine Patients [*n*]	Controls [*n*]	*p*-Value
*rs10405121*	AA (abnormal)	20	9	0.94173
	GA (heterozygous)	40	25	0.32201
	GG (normal)	40	16	0.43783
*rs894252513*	CA (abnormal)	1	0	1.0000
	CC (normal)	99	50	1.0000
*rs1012663275*	CC (abnormal)	1	2	0.53619
	AC (heterozygous)	5	14	**0.00019**
	AA (normal)	94	34	**0.00006**

**Table 4:** CADD scores for three novel intronic *CACNA1A* variants.

Variant (hg38)	Nucleotide Change	CADD PHRED Score	Predicted Effect
*chr19:13228374*	G > C	0.921	Likely benign
*chr19:13228428*	G > C	17.06	Possibly functional/deleterious
*chr19:13228348*	A > T	6.464	Possibly mild regulatory effect

**Table 5:** Demographic data of the study group and the control group.

Migraine (*n* = 100)			Controls (*n* = 50)
	**MO ^1^ (*n* = 50)**	**MA ^2^ (*n* = 50)**	
**Sex**			
Male	3	5	6
Female	47	45	44
Age	35.44 ± 13.33	36.64 ± 13.90	34.12 ± 13.90
**Family history of migraine**			
Yes	46	36	not applicable
No	4	14	
**Age of onset**			
1–18	31	31	not applicable
18–45	16	18	
>45	3	1	

^1^ Migraine without aura. ^2^ Migraine with aura.

## Data Availability

Data is contained within the article. Further inquiries can be directed to the corresponding author(s).

## Abbreviations

The following abbreviations are used in this manuscript:

WHO	World Health Organization
YLDs	Years lived with disability
MA	Migraine with aura
MO	Migraine without aura
CSD	Cortical spreading depression
CACNA1A	Calcium voltage-gated channel subunit alpha 1
ATP1A2	ATPase Na^+^/K^+^ transporting subunit alpha 2
SCN1A	Sodium voltage-gated channel alpha subunit 1
FHM	Familial hemiplegic migraine
FHM1	Familial hemiplegic migraine type 1
SCA6	Spinocerebellar ataxia type 6
ICHD-3	International Classification of Headache Disorders, 3rd edition
PUMS	Poznan University of Medical Sciences
EDTA	Disodium edetate
HRMA	High-resolution melt analysis
